# Tumour cell‐intrinsic CTLA4 regulates PD‐L1 expression in non‐small cell lung cancer

**DOI:** 10.1111/jcmm.13956

**Published:** 2018-10-30

**Authors:** Huijun Zhang, Pranabananda Dutta, Jinguo Liu, Nafiseh Sabri, Yuanlin Song, Willis X. Li, Jinghong Li

**Affiliations:** ^1^ Division of Pulmonary and Critical Care Medicine Department of Medicine University of California San Diego La Jolla California; ^2^ Department of Pulmonary Medicine Zhongshan Hospital Fudan University Shanghai China

**Keywords:** CTLA4, EGFR, NSCLC, PD‐1, PD‐L1

## Abstract

Cytotoxic T lymphocyte antigen 4 (CTLA4) and programmed cell death protein 1 (PD‐1) are immune checkpoint proteins expressed in T cells. Although CTLA4 expression was found in multiple tumours including non‐small cell lung cancer (NSCLC) tissues and cells, its function in tumour cells is unknown. Recently, PD‐1 was found to be expressed in melanoma cells and to promote tumorigenesis. We found that CTLA4 was expressed in a subset of NSCLC cell lines and in a subgroup of cancer cells within the lung cancer tissues. We further found that in NSCLC cells, anti‐CTLA4 antibody can induce PD‐L1 expression, which is mediated by CTLA4 and the EGFR pathway involving phosphorylation of MEK and ERK. In CTLA4 knockout cells, EGFR knockout cells or in the presence of an EGFR tyrosine kinase inhibitor, anti‐CTLA4 antibody was not able to induce PD‐L1 expression in NSCLC cells. Moreover, anti‐CTLA4 antibody promoted NSCLC cell proliferation in vitro and tumour growth in vivo in the absence of adaptive immunity. These results suggest that tumour cell‐intrinsic CTLA4 can regulate PD‐L1 expression and cell proliferation, and that anti‐CTLA4 antibody, by binding to the tumour cell‐intrinsic CTLA4, may result in the activation of the EGFR pathway in cancer cells.

## 1 INTRODUCTION

Anti‐cytotoxic T lymphocyte antigen 4 (CTLA4) antibodies have been shown to reverse T cell anergy leading to anti‐tumour responses.^1^ Anti‐CTLA4 therapy was the first FDA approved immunotherapy and has achieved significant therapeutic effects in metastatic melanoma.^2^ In non‐small cell lung cancer (NSCLC), anti‐CTLA4 therapy only showed moderate therapeutic effects when combined with chemotherapy.^3^ On the other hand, anti‐programmed cell death protein 1 (PD‐1) antibody has generated many exciting data from recent clinical trials in several cancers including NSCLC.^4^, ^5^, ^6^ Anti‐PD‐1 antibody blocks the interaction between PD‐1 and its ligand, programmed death‐ligand 1 (PD‐L1) and PD‐L2, leads to the reversal of previously exhausted immune responses.^7^ Combined anti‐CTLA4 and anti‐PD‐1 antibodies treatment was approved in metastatic melanoma with better efficacy compare with single agent.^8^, ^9^ The rationale of the better efficacy is based on the observation that anti‐CTLA4 targets the circulating T cells and anti‐PD‐1 targets the tumour infiltrated T cells.^10^ Together, they function at different steps to enhance T cell activation. Despite many progresses, only a fraction of patients with solid tumours benefits from anti‐CTLA4 or anti‐PD‐1 treatment.^5^, ^7^ Currently, PD‐L1 is the predictive biomarker for the responsiveness of anti‐PD‐1 treatment with limited success.^11^

It has been reported that CTLA4 and PD‐1 are expressed in NSCLC tumour tissues and cell lines, but not in normal bronchial epithelium.^12^, ^13^, ^14^ CTLA4 is expressed in many cell lines from variety of solid tumours. Treatment with CTLA4 ligands CD80/CD86 induces apoptosis with activation of caspase‐8 and caspase‐3.^13^ CTLA4 expression levels in NSCLC cancer tissues have been studied for its relevance in prognosis.^12^ These data indicate that tumour cell‐intrinsic CTLA4 may play a role in tumorigenesis. Recently, melanoma cells were found to have tumour cell‐intrinsic expression of PD‐1. The cell‐intrinsic PD‐1 engages with its ligand, PD‐L1, to promote tumorigenesis and modulate downstream mTOR signalling, in the absence of adaptive immunity.^15^

The interactions among CTLA4, PD‐1/PD‐L1 and oncogenic mutations are under investigation. In melanoma cells resistant to combined anti‐CTLA4 and radiation treatment, they have elevated PD‐L1 expression.^16^ CD4+ T cells from bladder cancer patients receiving anti‐CTLA4 treatment had markedly increased production of IFN‐γ.^17^ IFN‐γ is known to induce PD‐L1 expression.^18^ Oncogenic EGFR activation has been found to up‐regulate PD‐1 and PD‐L1.^19^, ^20^ PD‐L1 expression was associated with adenocarcinoma and EGFR mutations.^14^

In order to understand whether CTLA4 is expressed in NSCLC, whether tumour cell‐intrinsic CTLA4 plays a role in tumorigenesis, we examined the CTLA4, PD‐1, PD‐L1 expression levels in multiple NSCLC cell lines with different oncogenic mutations and in the tissue samples from NSCLC patients. We found that CTLA4 was expressed in a subset of NSCLC cell lines and in a subgroup of cancer cells within the lung cancer tissues. We further found that tumour cell‐intrinsic CTLA4 regulates PD‐L1 expression and cell proliferation via the EGFR pathway.

## 2 MATERIALS AND METHODS

### 2.1 Cell culture and reagents

Human NSCLC cell lines A549, H460, HCC827, H1975, H1650, H661 cells were obtained from the American Type Culture Collection (ATCC, Manassas, VA, USA). A549 CTLA4 knockout or EGFR knockout cells were generated by CRISPR/Cas9 method. Stable A549 CTLA4 overexpression cells were generated by Fugene reagent (Promega, Madison, WI, USA). Anti‐CTLA4 and anti‐PD‐1 antibodies for Western blot and Immunohistochemistry (IHC) were obtained from Abcam (Cambridge, MA, USA). Anti‐CTLA4 and anti‐PD‐1 antibodies for in vivo treatment were obtained from Bio X Cell (West Lebanon, NH, USA). Anti‐PD‐L1, EGFR, pEGFR, MEK, pMEK, pERK, pS6 antibodies were obtained from Cell Signaling (Danvers, MA, USA). IFN‐γ was obtained from Sigma‐Aldrich (St. Louis, MO, USA). This study has collected FFPE cancer tissue from NSCLC in patients who underwent surgical procedures at Zhongshan Hospital. The study protocol was approved by the internal review board of Zhongshan Hospital. All patients gave signed, written, informed consent on the use of clinical specimens for medical research.

### 2.2 Western blot

Cells were allowed to proliferate to 60%‐70% of maximum occupancy on tissue culture plastic, and treated as indicated. For the excised tumour, the tissues were homogenized on ice in cell lysis buffer. The lysates containing equal amounts of protein were loaded on a 10% SDS/PAGE gel. Antibodies for Western blot are as indicated.

### 2.3 Immunohistochemistry and immunofluorescence staining

Formalin‐fixed paraffin embedded specimens were serially sectioned at 5 um intervals and mounted on glass slides. IHC for CTLA4, PD‐1, PD‐L1 were performed using the antibodies as indicated. NSCLC cells were grown on chamber slides and were fixed with 4% formaldehyde, followed by primary and secondary antibody. Cells were counterstained with DAPI to show the nuclei.

### 2.4 Generate CRISPR/Cas9 knockout NSCLC cells

We obtained the pSpCas9‐2A‐Puro (PX459) v2.0 Plasmid from Addgene (Cambridge, MA, USA). DNA Oligos were synthesized and ligated into PX459 according to the protocol.^21^ Plasmids were transfected into A549 cells and stable cell lines were selected using puromycin to generate A549 EGFR KO cells and A549 CTLA4 KO cells. The effectiveness of knockout was determined by Western blot. Oligo sequences for sgRNA are as the following: EGFR_sg_F1: 5‐CACCgTGAGCTTGTTACTCGTGCCT‐3; EGFR_sg_R1: 5‐AAACAGGCACGAGTAACAAGCTCAc‐3; EGFR_sg_F2: 5‐CACCgGAGTAACAAGCTCACGCAGT‐3; EGFR_sg_R2: 5‐AAACACTGCGTGAGCTTGTTACTCc‐3; CTLA4_sg_F1: 5‐CACCgGTACCCACCGCCATACTACC‐3; CTLA4_sg_R1: 5‐AAACGGTAGTATGGCGGTGGGTACc‐3; CTLA4_sg_F2: 5‐CACCgTTGCCTATGCCCAGGTAGTA‐3; CTLA4_sg_R2: 5‐AAACTACTACCTGGGCATAGGCAAc‐3.

### 2.5 Cell proliferation MTT assay

Cell proliferation was determined by 3‐(4,5‐dimethylthiazol‐2‐yl)‐2,5‐diphenyltetrazolium bromide (MTT) assay. Cells (15 × 10^3^) were incubated in 96‐well plates for 24 hours in complete medium before the addition of treatment as indicated. MTT solution (1 mg/mL; Sigma‐Aldrich) was added to a final concentration of 0.4 mg/mL, and plates were incubated at 37°C for 3 hours. The absorbance was recorded at 540 nm. Statistical analyses were performed using Graph Pad Prism software (GraphPad Software, La Jolla, CA, USA).

### 2.6 Xenograft experiments

All studies were conducted in accordance with the University of California San Diego Institutional Animal Care and Used Committee (IACUC) and the National Institutes of Health guideline for the care and use of laboratory animals. Nude mice (nu/nu) were obtained from Charles River Laboratories (Wilmington, MA, USA). At day 0, a total of 5.0 × 10^6^ cells (A549) were implanted subcutaneously into the right flank of 6‐week‐old nude mice. At day 7, the average tumour size reached 50‐100 mm^3^, mice were randomized and divided into different treatment groups (n = 8 for each group). Anti‐CTLA4 (clone BNI3), anti–PD‐1 (clone J116) or control IgG1 antibodies were administered at 200 μg in 100 μL of PBS by intraperitoneal injection. The injections were done twice weekly. The tumour sizes were measured twice weekly. At day 42, after tumour diameters are measured, animals were sacrificed and tumours were excised. Statistical analyses were performed using Graph Pad Prism software.

## 3 RESULTS

### 3.1 CTLA4 and PD‐L1 are expressed in NSCLC cell lines and tissue samples

**Figure 1:**
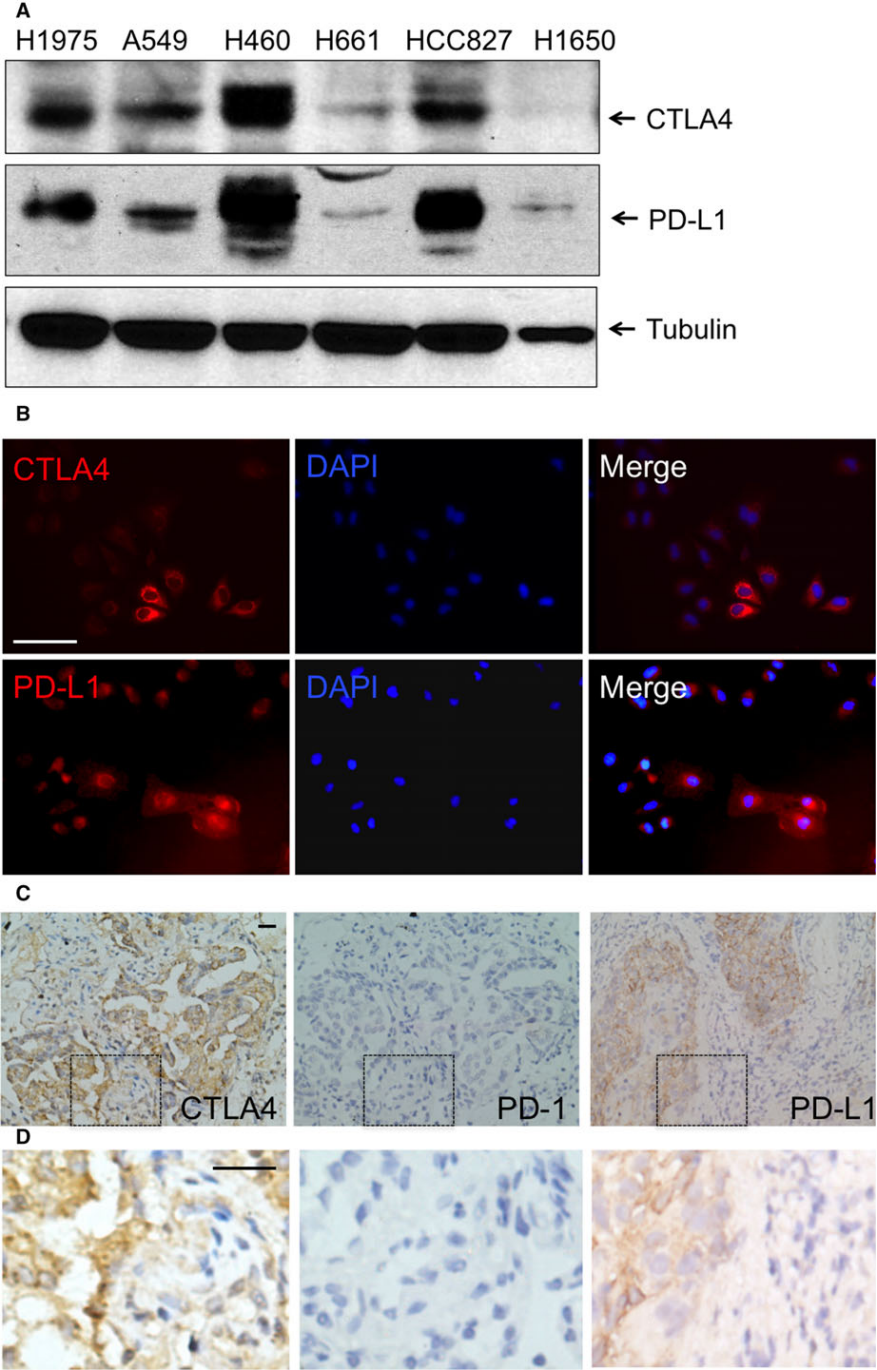
CTLA4 and PD‐L1 expression in NSCLC cells. A, CTLA4 is expressed at various levels in NSCLC cell lines. PD‐L1 is expressed in all NSCLC cell lines. Individual cell line is as marked. B, CTLA4 and PD‐L1 are localized in the cytoplasm of NSCLC cells, with high concentration of the protein is found to surround the nuclear membrane. A549 cells are shown. C, NSCLC patient tissue samples with mutations in K‐Ras (n = 3), in EGFR (n = 3), without mutations in these two genes (n = 3) were stained for CTLA4, PD‐1 and PD‐L1, respectively. All samples regardless of K‐Ras and EGFR mutation status showed similar expression: CTLA4 is expressed not only in tumour‐infiltrated lymphocytes, but also lung cancer cells (left). PD‐1 expression is undetectable in the tissue samples we tested (middle). PD‐L1 is expressed in a subgroup of cancer cells within the lung cancer tissues from NSCLC patients (right). Representative images are shown. D, Higher magnifications of regions boxed with dashed lines in C. Scale bars are 50 μm

We first examined the protein expression of CTLA4, PD‐1 and PD‐L1 in a few NSCLC cell lines. By Western blotting, we found that CTLA4 and PD‐L1 are expressed at various levels in NSCLC cell lines. Both CTLA4 and PD‐L1 are expressed in A549, H460, HCC827, H1975 cells at relatively high levels. They are expressed in H661 cells at very low levels, and are not expressed at H1650 cells (Figure 1A). PD‐1 is expressed at low levels in all these cells (data not shown). In normal bronchial epithelial cells, BEAS‐2B, there is no detectable expression of CTLA4 or PD‐1 (data not shown). By immunostaining, we again found that CTLA4 and PD‐L1 are expressed in NSCLC cells (Figure 1B). In the lung cancer tissue samples from NSCLC patients, CTLA4 and PD‐L1, are expressed not only in tumour‐infiltrated lymphocytes, but also lung cancer cells (Figure 1C,D). On the other hand, PD‐1 is barely detectable in the tissue samples from NSCLC patients (Figure 1C and D).

### 3.2 Anti‐CTLA4 increases PD‐L1 expression in NSCLC cell lines

**Figure 2:**
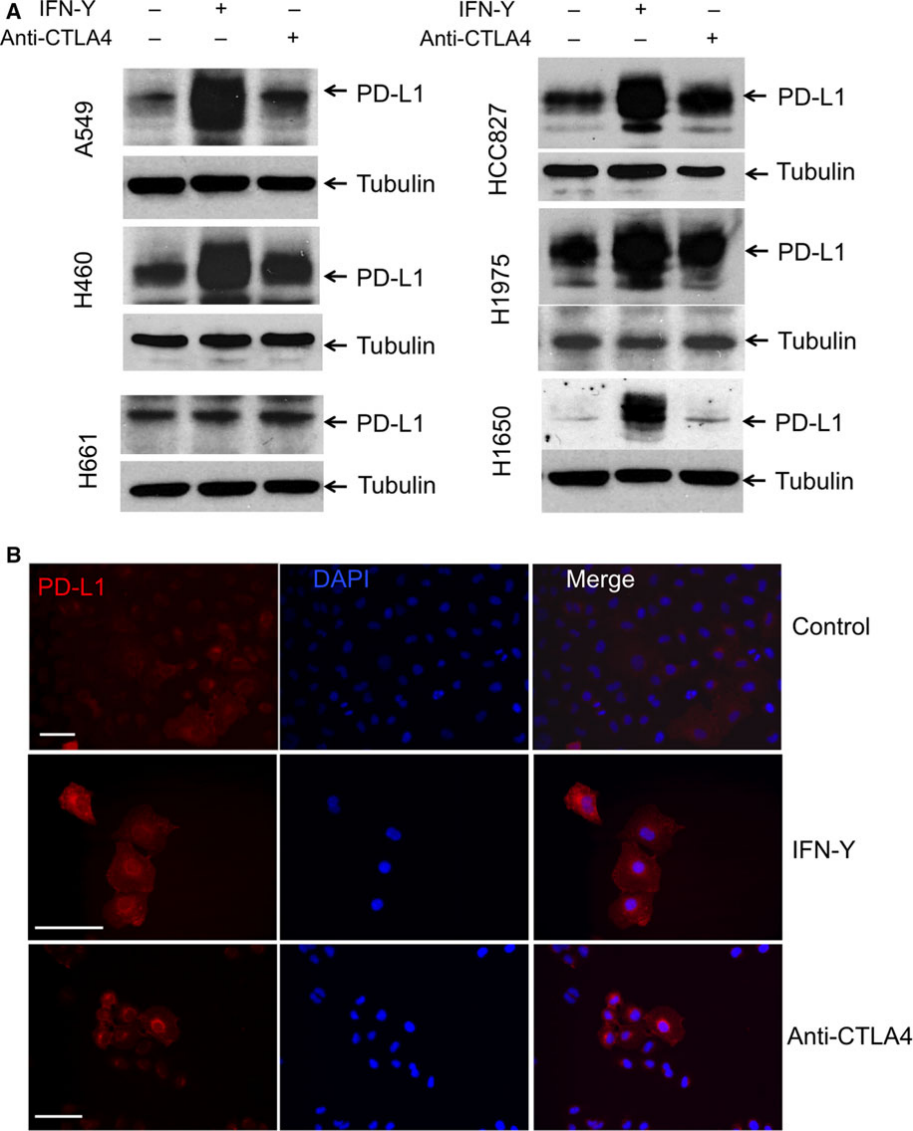
Effects of IFN‐γ and anti‐CTLA4 antibody treatment on PD‐L1 expression in NSCLC cells. A, The indicated cells were treated with IFN‐γ (100 ng/mL) or anti‐CTLA4 antibody (10 μg/mL) for 24 h. The anti‐CTLA4 antibody induced up‐regulation of PD‐L1 was observed in A549, H460, HCC827 and H1975, but not in H661 and H1650 cells. B, IFN‐γ or Anti‐CTLA4 antibody treatment increases cellular PD‐L1 (red) in A549 cells. Scale bars are 50 μm

The similar expression pattern of CTLA4 and PD‐L1 in lung cancer cells prompted us to investigate whether one protein affect the expression of the other. In melanoma, it has been shown that anti‐CTLA4 antibody treatment initially reduces tumour burden, but the resistant cancer cells have increased PD‐L1 expression.^16^ We found that anti‐CTLA4 antibody, when present in the culture media, increased PD‐L1 expression in NSCLC cell lines (Figure 2A). IFN‐γ, which is known to stimulate PD‐L1 expression,^18^ was used as a positive control. The anti‐CTLA4 antibody induced up‐regulation of PD‐L1 is observed in the cell lines with higher CTLA4 expression, such as in A549, H460, HCC827 and H1975, but not in those with low or no CTLA4 expression, such as H661 and H1650 (Figure 2A). These cells are EGFR wild‐type or EGFR activating mutations NSCLC cells. Using fluorescent immune staining of A549 cells, we observed that, while IFN‐γ induced PD‐L1 expression in all cells, anti‐CTLA4 did so in a subset of cells (Figure 2B).

To understand if the regulation is at transcriptional level, we measured PD‐L1 mRNA levels in cells after treatment with IFN‐γ or anti‐CTLA4. We found that A549 cells, but not H661 cells, responded to the treatment by increasing PD‐L1 mRNA (Figure S1), consistent with changes in protein levels measured by Western blots. Thus, anti‐CTLA4 seems to increase PD‐L1 transcription in a subset of NSCLC cells.

### 3.3 Anti‐CTLA4 induced up‐regulation of PD‐L1 is mediated by EGFR pathway

**Figure 3:**
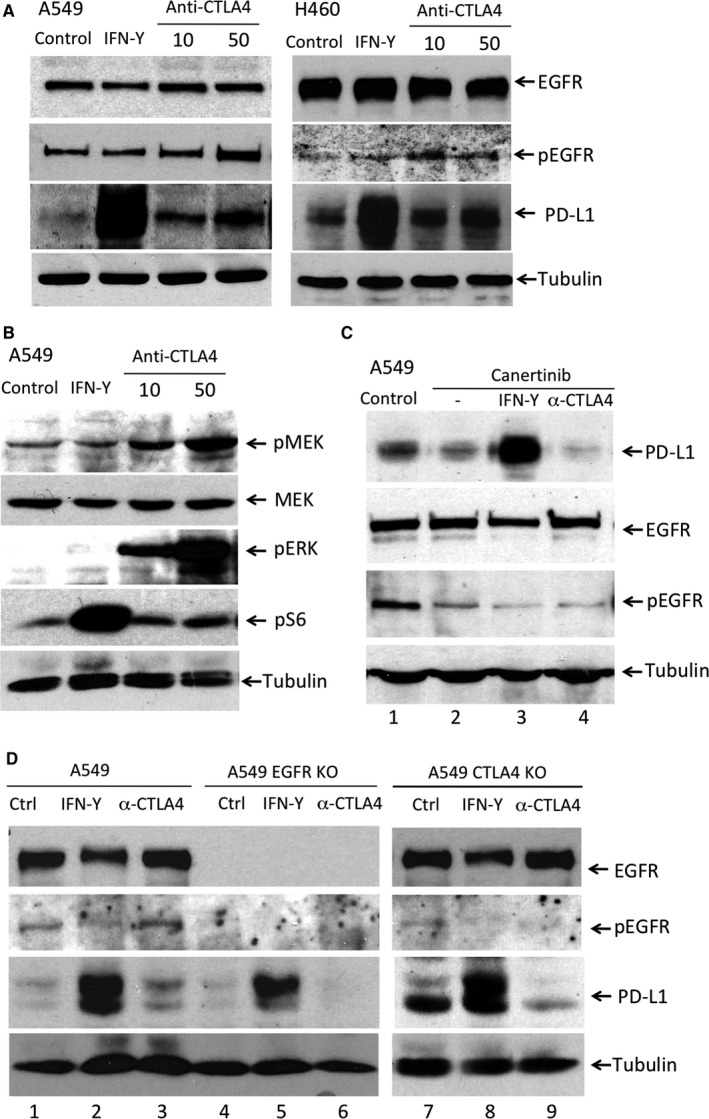
Anti‐CTLA4 induced PD‐L1 expression is EGFR pathway dependent in NSCLC cells. A, The A549 cells or H460 cells were treated with IFN‐γ (100 ng/mL) or anti‐CTLA4 antibody (10, 50 μg/mL) for 24 h. Anti‐CTLA4 antibody increases pEGFR, but not total EGFR, in a dose‐dependent manner. The increase of pEGFR is in parallel with the increase of PD‐L1. B, Anti‐CTLA4 antibody increases pMEK, pERK levels, not the total MEK or pS6. IFN‐γ increases the pS6 levels, but not the pMEK or pERK levels. C, EGFR inhibitor, Canertinib, inhibits pEGFR and anti‐CTLA4 induced up‐regulation of PD‐L1. Canertinib has no effect on IFN‐γ induced PD‐L1 expression. D, In A549 EGFR knockout (KO) cells, anti‐CTLA4 is unable to induce PD‐L1 expression (lane 6). In A549 CTLA4 KO cells, anti‐CTLA4 induced up‐regulation of PD‐L1 is diminished (lane 9). IFN‐γ induced PD‐L1 expression is not affected by EGFR KO (lane 5) or CTLA4 KO (lane 8)

We then investigated the mechanisms that mediated the anti‐CTLA4 induced PD‐L1 expression. Based on our findings that both CTLA4 and PD‐L1 are expressed at higher levels in EGFR wild‐type or EGFR activating mutations NSCLC cells (Figure 2A), we focused on determining the EGFR activation status. We found that the level of phosphorylated EGFR (pEGFR) was indeed increased after anti‐CTLA4 antibody treatment in a dose‐dependent manner, whereas the total EGFR levels were not changed (Figure 3A). IFN‐γ treatment, on the other hand, did not alter pEGFR levels (Figure 3A).

To further substantiate this finding, we examined the levels of EGFR downstream components, phosphorylated MEK (pMEK) and phosphorylated ERK (pERK). We found that anti‐CTLA4 also increased pMEK, pERK levels in a dose‐dependent manner (Figure 3B). In contrast, IFN‐γ increases phosphorylated S6 Ribosomal Protein (pS6) levels without altering pMEK and pERK levels (Figure 3B). Next, we examined the effects of EGFR inhibition or EGFR knockout on anti‐CTLA4 induced PD‐L1 expression. We found that the EFGR inhibitor Canertinib suppressed pEGFR levels and also suppressed anti‐CTLA4 induced PD‐L1 expression levels (Figure 3C, lane 4). Of note, Canertinib has no effect on IFN‐γ induced PD‐L1 expression (Figure 3C, lane 3). We then generated A549 EGFR knockout (KO) cells and A549 CTLA4 KO cells by CRISPR/Cas9. In EGFR KO cells, anti‐CTLA4 is unable to induce PD‐L1 expression (Figure 3D, lane 6), while IFN‐γ induced PD‐L1 expression is intact (Figure 3D, lane 5). In CTLA4 KO cells, the anti‐CTLA4 induced up‐regulation of PD‐L1 is diminished too (Figure 3D, lane 9). Again, IFN‐γ induced PD‐L1 expression is intact (Figure 3D, lane 8).

### 3.4 Effects of anti‐CTLA4 antibody on cancer cell proliferation in vitro and tumour growth in vivo

**Figure 4:**
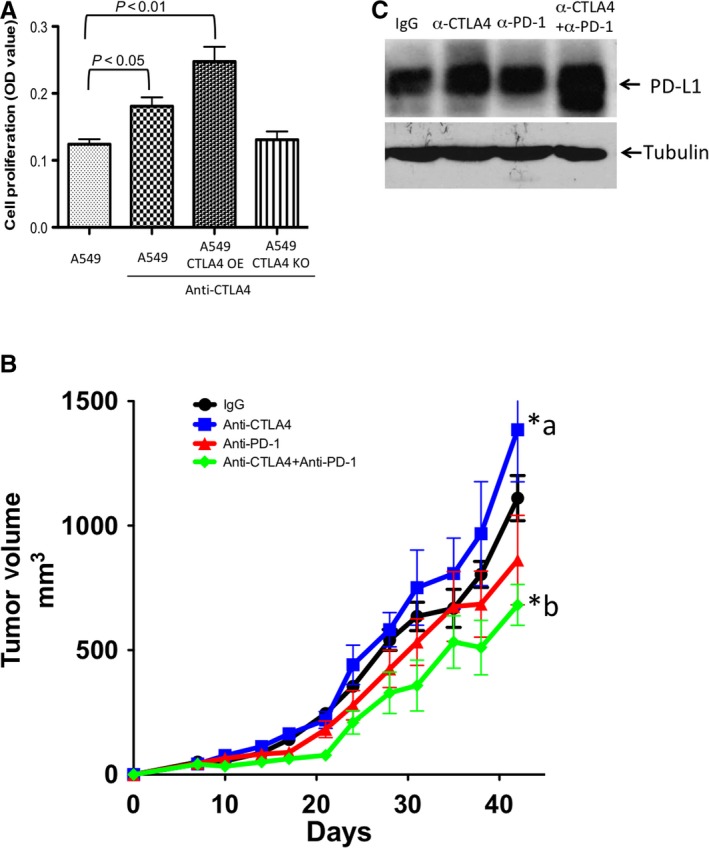
Effects of anti‐CTLA4 antibody on cell proliferation and tumour growth in the absence of adaptive immunity. A, In A549, A549 CTLA4 KO and A549 CTLA4 overexpression (OE) cells, anti‐CTLA4 antibody treatment promoted cell proliferation in A549 cells, more so in A549 CTLA4 OE cells, but not in A549 CTLA4 KO cells. Data are shown as means ± SEM (n = 10), compared with A549 control group, *P* < 0.05, *P* < 0.01 as marked. B, In nude mice xenograft experiments, anti‐CTLA4 antibody increases the xenograft tumour growth compared with control IgG treatment. Data are shown as means ± SEM (n = 8), compared with control IgG group, *a, *P* < 0.05. Anti‐PD‐1 antibody has negative effects on tumour growth but not significant. The combination of anti‐CTLA4 and anti‐PD‐L1 antibodies significantly decreased the tumour growth. Data are shown as means ± SEM (n = 8), compared with control IgG group, *b, *P* < 0.05. C. In the excised xenograft tumour, anti‐CTLA4 antibody increases the PD‐L1 expression. The combination of anti‐CTLA4 and anti‐PD‐1 antibodies further increases PD‐L1 expression in the tumour tissue

Based on our finding that anti‐CTLA4 antibody activates the EGFR pathway in NSCLC cells, we investigated its effects on cell proliferation. We used MTT assay in A549 cells, A549 CTLA4 KO cells and A549 CTLA4 overexpression (OE) cells. We found that anti‐CTLA4 antibody treatment promoted cell proliferation in A549 cells (Figure 4A), more so in A549 CTLA4 OE cells, but not in A549 CTLA4 KO cells (Figure 4A). We next examined the effects of anti‐CTLA4 and anti‐PD‐1 treatment on xenograft tumour growth in nude mice. Nude mice do not have T cells, allowing for evaluating the function of tumour cell‐intrinsic CTLA4 in the absence of adaptive immunity. We found that anti‐CTLA4 antibody increased the xenograft tumour growth compared with control IgG treatment (Figure 4B). Anti‐PD‐1 antibody decreases the xenograft tumour growth. Combination of anti‐CTLA4 and anti‐PD‐L1 antibodies further decreases the tumour growth (Figure 4B). In the excised tumour tissue, anti‐CTLA4 antibody increases the PD‐L1 expression, whereas anti‐PD‐1 antibody does not have significant effects on PD‐L1 expression. Combination of anti‐CTLA4 and anti‐PD‐1 antibodies increases PD‐L1 expression in the tumour tissue (Figure 4C).

## 4 DISCUSSION

Our current knowledge about CTLA4 and PD‐1 are mostly based on their functions in T cells.^22^ Although CTLA4 expression levels have been studied for the relevance of prognosis in NSCLC,^12^ and CTLA4 was found to be expressed in multiple tumours including NSCLC,^12^, ^13^, ^23^, ^24^ no further studies were done to reveal its function in tumour cells. We demonstrated that CTLA4 was expressed in a subset of NSCLC cell lines and in a subgroup of cancer cells within the lung cancer tissues. The expression levels of CTLA4 and PD‐L1 correlate with each other and they are both higher in EGFR wild‐type or EGFR activating mutations NSCLC cells.

It has been reported that the EGFR and Hippo/YAP pathways are involved in regulation of PD‐L1 expression.^14^, ^19^, ^25^, ^26^, ^27^ We provided the evidence that anti‐CTLA4 antibody could induce PD‐L1 expression in NSCLC cells. However, anti‐CTLA4 was unable to induce PD‐L1 expression in CTLA4 KO or EGFR KO cells, or in the presence of an EGFR‐specific tyrosine kinase inhibitor. CTLA4 and EGFR are both required for anti‐CTLA4 induced PD‐L1 up‐regulation in NSCLC cells. These results indicate that anti‐CTLA4 antibody binds to the tumour cell‐intrinsic CTLA4 on the cell surface, resulting in activation of the EGFR pathway, which induces PD‐L1 expression. Anti‐CTLA4 and IFN‐γ use different pathways to regulate PD‐L1 expression, with anti‐CTLA4 activating the EGFR pathway and IFN‐γ functioning through the mTOR/S6 pathway.^28^

We found that anti‐CTLA4 antibody could promote NSCLC cell proliferation in vitro. The cell proliferation was further increased in A549 CTLA4 OE cells, but was diminished in A549 CTLA4 KO cells. The xenograft tumour growth in vivo studies were done in the absence of adaptive immunity. Anti‐CTLA4 antibody again promoted NSCLC tumour growth. These data suggest that tumour cell‐intrinsic CTLA4 plays a role in tumorigenesis. This may explain the lack of efficacy of anti‐CTLA4 treatment in NSCLC. NSCLC patients received anti‐CTLA4 antibody may have beneficial effects from the circulating T cells activation, but anti‐CTLA4 antibody also promotes NSCLC cell proliferation via the tumour cell‐intrinsic CTLA4. Combination of anti‐CTLA4 and anti‐PD‐1 antibodies have better efficacy than anti‐PD‐1 antibody alone. These results were not explained by the previous rationale that better efficacy was due to anti‐CTLA4 and anti‐PD‐1 acting at different steps to enhance T cell activation,^10^ because the nude mice are lacking of immune cells. In the excised tumour tissue, PD‐L1 expression was increased in anti‐CTLA treatment group. The combination of anti‐CTLA4 and anti‐PD‐1 antibodies further increased PD‐L1 expression. These data suggest that the increased PD‐L1 expression in the tumour cells may provide the target for anti‐PD‐1 antibody for potentiating the tumour suppression.

Our results advanced our current understanding of the function of CTLA4 in NSCLC. The tumour cell‐intrinsic expression of CTLA4 has a different function than that of the checkpoint protein in T cells. Anti‐CTLA4 antibody binds with the tumour cell‐intrinsic CTLA4 and activates the EGFR pathway to induce PD‐L1 expression. A panel of CTLA4, PD‐1, PD‐L1, EGFR expression levels may better predict the responsiveness of anti‐PD‐1 treatment compared with the PD‐L1 as a single predictive biomarker. Tumour cell‐intrinsic CTLA4 expression provides us new therapeutic target for NSCLC treatment. In NSCLC with wild‐type EGFR and high expression levels of CTLA4, anti‐CTLA4 antibody may induce PD‐L1 expression to potentiate the efficacy of anti‐PD‐1 treatment. Other molecules or peptides that can activate the tumour cell‐intrinsic CTLA4 will be worthwhile to investigate.

## CONFLICT OF INTEREST

The authors confirm that there are no conflicts of interest.

## AUTHOR CONTRIBUTION

JL and WXL designed the research studies, and wrote the manuscript; HZ, PD, JGL, NS conducted experiments and acquired data; HZ, YS, JL, WXL analysed the data.
