# Supplementary material for: Tumour cell‐intrinsic CTLA4 regulates PD‐L1 expression in non‐small cell lung cancer
Source: J Cell Mol Med. 2018 Oct 30;23(1):535–42. doi: 10.1111/jcmm.13956 (PMC6307812; doi:10.1111/jcmm.13956)
Supplement: Supplementary file 1 [file JCMM-23-535-s001.pdf]

Supplementary Information

Tumor cell-intrinsic CTLA4 regulates PD-L1 expression in non-small cell lung cancer

Huijun Zhang*†, Pranabananda Dutta*, Jinguo Liu†, Nafiseh Sabri*, Yuanlin Song†, Willis X. Li*, Jinghong Li*§

*Division of Pulmonary and Critical Care Medicine

Department of Medicine, University of California San Diego, La Jolla, CA 92093

†Department of Pulmonary Medicine

Zhongshan Hospital, Fudan University, Shanghai, China

Running title: Tumor cell-intrinsic CTLA4 in NSCLC

Key words: CTLA4, PD-1, PD-L1, EGFR, NSCLC

This work was supported by: American Thoracic Society (ATS) Foundation (JL)

§Correspondence to:

Jinghong Li, M.D., Ph.D.

Division of Pulmonary and Critical Care Medicine

Department of Medicine, University of California San Diego, La Jolla, CA 92093

jinghongli@ucsd.edu

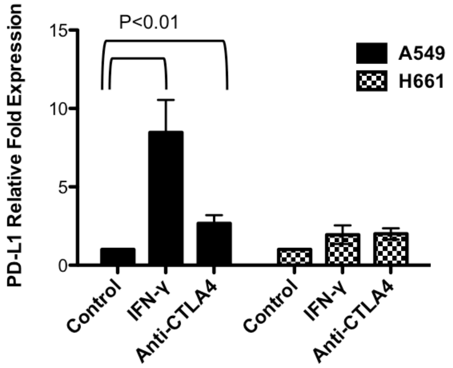

Figure S1. PD-L1 mRNA level changes in response to stimuli

NSCLC A549 or H661 cells were treated with IFN-γ (100ng/mL) or anti-CTLA4 antibody (10 μg/ml) for 24 hours. Total mRNA was isolated with the level of PD-L1 mRNA was measured with real-time PCR in triplicates. Data are shown as means ± SEM. $p<0.01$ (Student's t test), compared with untreated controls, are indicated.

Methods: RNA was isolated with TRI reagent (Sigma-Aldrich). Isolated RNA was reverse-transcribed with SuperScript II RNase reverse transcriptase (Invitrogen, Carlsbad, CA). Specific primer pairs for PD-L1 and GAPDH were used. PD-L1 primers are TGTACCGCTGCATGATCAG and AGTTCATGTTCAGAGGTGACTG. Direct detection of the PCR product was monitored by measuring the increase in fluorescence caused by the binding of SYBR Green to dsDNA (Applied Biosystems) per well during 40 cycles. The cycle threshold (*C*t) method was used to assess changes in mRNA expression: fold change = $2^{-\Delta\Delta Ct}$

.
